# The AMPK pathway in fatty liver disease

**DOI:** 10.3389/fphys.2022.970292

**Published:** 2022-08-25

**Authors:** Chunqiu Fang, Jianheng Pan, Ning Qu, Yuting Lei, Jiajun Han, Jingzhou Zhang, Dong Han

**Affiliations:** ^1^ College of Pharmacy, Changchun University of Chinese Medicine, Changchunn, China; ^2^ College of Traditional Medicine, Changchun University of Chinese Medicine, Changchun, China

**Keywords:** lipid accumulation, AMPK signaling pathway, alcoholic fatty liver, non-alcoholic fatty liver, lipid metabolism

## Abstract

Lipid metabolism disorders are the primary causes for the occurrence and progression of various liver diseases, including non-alcoholic fatty liver disease (NAFLD) and alcoholic fatty liver disease (AFLD) caused by a high-fat diet and ethanol. AMPK signaling pathway plays an important role in ameliorating lipid metabolism disorders. Progressive research has clarified that AMPK signal axes are involved in the prevention and reduction of liver injury. Upregulation of AMK can alleviate FLD in mice induced by alcohol or insulin resistance, type 2 diabetes, and obesity, and most natural AMPK agonists can regulate lipid metabolism, inflammation, and oxidative stress in hepatocytes, consequently regulating FLD in mice. In NAFLD and AFLD, increasing the activity of AMPK can inhibit the synthesis of fatty acids and cholesterol by down-regulating the expression of adipogenesis gene (FAS, SREBP-1c, ACC and HMGCR); Simultaneously, by increasing the expression of fatty acid oxidation and lipid decomposition genes (CPT1, PGC1, and HSL, ATGL) involved in fatty acid oxidation and lipid decomposition, the body’s natural lipid balance can be maintained. At present, some AMPK activators are thought to be beneficial during therapeutic treatment. Therefore, activation of AMPK signaling pathway is a potential therapeutic target for disorders of the liver. We summarized the most recent research on the role of the AMPK pathway in FLD in this review. Simultaneously, we performed a detailed description of each signaling axis of the AMPK pathway, as well as a discussion of its mechanism of action and therapeutic significance.

## Introduction

The liver, as the body’s largest digestive gland, performs essential functions such as secreting bile, regulating lipid metabolism, storing glycogen, and decomposing sugar (Zhou et al., 2022). In addition, the liver is also considered to be the master regulator of lipid homeostasis, responsible for coordinating fatty acid uptake, synthesis and oxidative decomposition, lipid export and redistribution. To maintain liver lipid homeostasis, these processes are complex regulated by hormones, nuclear receptors and transcription factors under physiological conditions. Unfortunately, if the balance of lipid metabolism in the liver was disturbed, it would lead to abnormal accumulation of lipid, and even induce oxidative stress, which would damage the liver and further encourage the incidence and development of FLD (Carotti et al., 2020). Fat accumulation is a key cause of induction of multitude liver diseases, including non-alcoholic fatty liver disease (NAFLD) and alcoholic fatty liver disease (AFLD) caused by a high-fat diet and ethanol consumption (Zhou et al., 2017; Carotti et al., 2020). It will further lead to liver fibrosis, cirrhosis, and even primary liver cancer.

**FIGURE 1:**
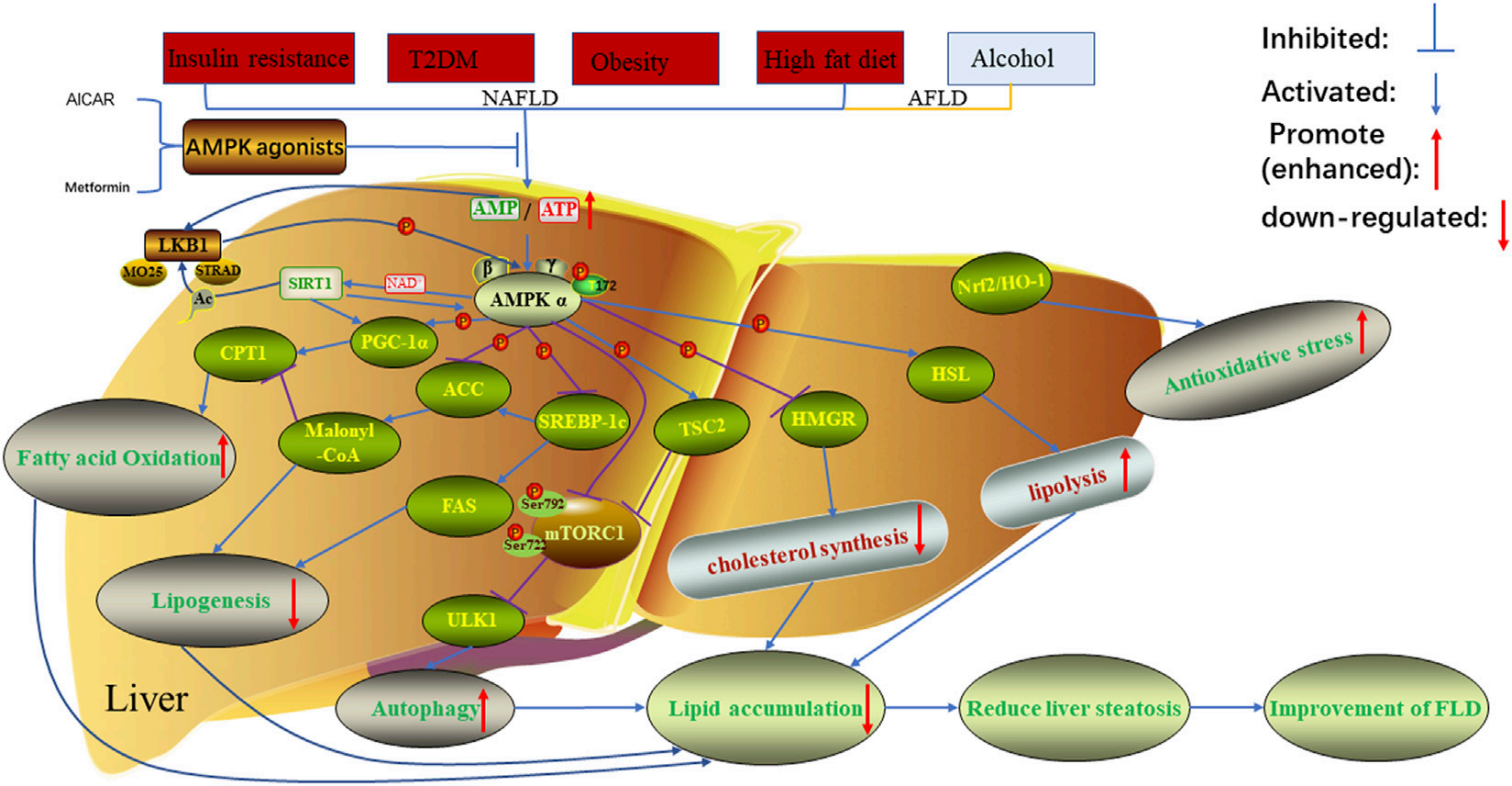
Regulation of AMPK on lipid metabolism in FLD. In the case of non-alcoholic fatty liver disease caused by insulin resistance, type 2 diabetes, obesity, high-fat diet and alcoholic fatty liver disease caused by alcohol, the related proteins in AMPK signaling pathway will be inhibited or promoted, resulting in increased lipid accumulation and decreased fatty acid oxidation. This was reversed when AMPK was treated with AMPK agonists. AMPK, AMP-activated protein kinase; AICAR, 5-aminoimidazole-4-carboxamide ribonucleotide; LKB1, Liver kinase B1; the auxiliary subunit STRAD, STE20 related adaptor protein, and MO25, Mouse protein 25; Sirt1, Silent mating type information regulation two homolog1; CPT1, carnitine acyltransferase one; PGC-1α, peroxisome proliferator-activated receptor *γ* co-activator -1α; ACC, acetyl-CoA carboxylase; SREBP-1c, Sterol regulatory element-binding protein 1c; FAS, fatty acid synthase; TSC2; mTORC1, mammalian target of rapamycin1; ULK1, unc51 like kinase one; HMGR, 3-hydroxy-3-methylglutaryl-CoA reductase; HSL, Hormone-Sensitive triglyceride lipase; Nrf2/HO-1, nuclear factor erythroid 2-related factor 2/heme oxygenase-1.

Lipid metabolism in adipose tissue of that body is regulated by several transcription factors, and the fat storage in the body depend on fat synthesis and catabolism, which are accomplished through the interaction between endogenous genes and external regulatory factors (Nielsen et al., 2014). When there is a lack of energy in the body, lipolysis is accelerated, which mainly releases FFA and glycerol into the blood through β-oxidation in mitochondria, and then enters other tissues through extensive circulation (Herzig and Shaw 2018). AMP-activated protein kinase (AMPK) is involved in multiple aspects of anti-lipid metabolism in cells and organisms, including oxidative decomposition of fatty acids and triglycerides, synthesis of fatty acids and triglycerides, to regulate cell metabolism and promote cell proliferation, and it is critical in the pathological mechanism of various FLD (Meng et al., 2019; Sharma et al., 2021) Figure 1. In addition, AMPK is an evolutionarily conserved serine/threonine protein kinase that is ubiquitous in eukaryotic cells, acting not only a sensor of cellular energy stress, but also a core hub for maintaining cellular energy homeostasis (Zhou et al., 2017; Gu et al., 2019). It is considered to be an important metabolic “master switch” that regulates the target kinases of lipid metabolism such as acetyl-CoA carboxylase (ACC) through phosphorylation (Ponnusamy et al., 2020). Recent studies have shown that AMPK exerted its ability to regulate lipid metabolism by enhancing fatty acid oxidation and autophagy, while inhibiting the production of cholesterol and fatty acids (Gu et al., 2018; Herzig and Shaw 2018). Previous study has explored the mechanism of L-theanine regulating lipid metabolism in SD rats *via* activating the AMPK signaling pathway (Lin et al., 2020). The results of their research showed that L-Theanine inhibited lipid synthesis by activating either LKB1-AMPK-SREBP-1c-FAS or LKB1-AMPK-SREBP-1c-ACC1 pathways Figure 1. The detection of related biochemical indicators showed that l-theanine could activate AMPK through LKB1, and significantly downregulate the expression levels of ACC phosphorylation and Sterol regulatory element-binding protein 1c (SREBP-1c). At the same time, l-theanine also significantly down-regulated the expression of FAS and HMGCR at the mRNA and protein levels. Additionally, detecting the phosphorylation levels of ACC1/2 and HMGCR, they found that the synthesis of lipids and cholesterol was inhibited, implying that AMPK was also involved in the LKB1-AMPK-ACC1 and LKB1-AMPK-HMGCR pathways to inhibit lipid and cholesterol synthesis. Thus, AMPK pathway may be a promising treatment option for FLD because it can maintain a steady state of lipid metabolism in response to various forms of stress. We review the most recent research on the role of the AMPK pathway in FLD. Furthermore, we also provided a detailed description of each signaling axis of the AMPK pathway, as well as a discussion of its mechanism of action and therapeutic significance.

## The structure of AMP-activated protein kinase and its effect on lipid metabolism

AMPK is a heterotrimeric complex composed of three subunits: a 63 KDaα subunit, a 38 KDaβ subunit and a 38 KDaγ subunit, where *α* is the catalytic subunit and *β* and *γ* are the regulatory subunits (Willows et al., 2017). The *α* subunit is made up of three domains from N-terminal to C-terminal, including a kinase domain, a self-inhibitory domain (AID) that can decrease AMPK activity at low levels of AMP and a carboxy-terminal domain (α-CTD) (Liang et al., 2017). Studies have shown that AMPK activity can be activated by phosphorylation of the Thr172 site of AMPKα subunit through upstream kinases (liver kinase B1 (LKB1) and CAMKKβ) and pharmacological activators (metformin, 5-aminoimidazole-4-carboxamide ribonucleotide (AICAR)) (Gai et al., 2020). In addition, AMPK activity was considerably impacted by variations in the intracellular AMP/ATP ratio. When AMP/ATP levels rose, AMPK was activated, inhibiting fat synthesis and promoting fatty acid oxidation (Hardie 2016; Ponnusamy et al., 2020). In mammals, these three subunits contain 7 subtypes, namely α1/α2, β1/β2, γ1/γ2/γ3 (Ross et al., 2016). Subunits α1, α2, γ1, and γ2 are mainly expressed in liver tissue (Liang et al., 2017). AMPK, as a significant metabolic regulatory component, can reflect the stress state of cells when the body is under oxidative stress and energy deficiency, and subsequently control the target proteins *via* phosphorylation, influencing lipid metabolism (Hardie 2015). Previous studies have confirmed that after AMPK was activated, it could regulate cell lipid metabolism by phosphorylating a series of metabolic proteins that affect fatty acid, cholesterol synthesis and fatty acid oxidation. For instance, AMPK inhibited the activity of 3-hydroxy-3-methylglutaryl-CoA reductase (HMGR) through phosphorylation, which plays a key role in regulating cholesterol synthesis (Loh et al., 2019) Figure 1. At the same time, AMPK can also phosphorylate HSL to increase its activity, thereby promoting the hydrolysis of fatty acylglycerol and cholesterol lipids (Kim et al., 2016) Figure 1. In addition, some researchers discovered that the following were the primary ways through which AMPK reduces liver fat accumulation (Smith et al., 2016; Li et al., 2019) Figure 1:1) Activated AMPK can phosphorylate ACC and inactivate it, preventing ACC dimerization and, as a result, reducing fatty acid synthesis; 2) Malonyl-CoA is not only a precursor of fatty acid synthesis, but also a potent inhibitor of carnitine acyltransferase1 (CPT1). AMPK phosphorylates ACC to inactivate it, reducing malonyl-CoA synthesis, which promotes the expression of CPT1, and thus increases fatty acid oxidation. In conclusion, AMPK can phosphorylate the expression of downstream key target proteins, reduce lipid accumulation, promote fatty acid oxidation, and inhibit the synthesis of cholesterol and fatty acids. As a result, the AMPK pathway may be a promising treatment option for FLD.

## The role of AMP-activated protein kinase-mediated signal axes in fatty liver

### Sirt1-liver kinase B1-AMP-activated protein kinase axis

As a tumor suppressor kinase, LKB1, also known as serine/threonine protein kinase 1, is widely distributed in a variety of embryonic and adult tissues. Through two mechanisms of phosphorylation and cellular localization, it participates in the signal regulation of intracellular fat differentiation and generation (Xu et al., 2020; Zhang et al., 2021). LKB1 is mainly located in the nucleus, but its functional performance is mainly triggered by the cytoplasmic parts, which plays an important role in the control and regulation of cell energy metabolism and apoptosis (Zuo et al., 2020). A member of the AMPKK family, LKB1 facilitates the movement of LKB1 from the nucleus to the cytoplasm by acting as an upstream kinase of AMPK, phosphorylating and activating AMPK (Zuo et al., 2020). Additionally, this activation is constitutive and can form a heterotrimer to phosphorylate and activate AMPK regardless of the intracellular AMP/ATP ratio by joining with the auxiliary subunits STRAD and MO25 (Ross et al., 2016; Herzig and Shaw 2018) Figure 1. Its downstream Silent information regulator 1 (sirt1) protein, peroxisome-proliferator-activated receptor co-activator-1α (PGC-1α), and a few transcription factors are then activated, helping to control the body’s lipid metabolism by preventing lipid synthesis, promoting fatty acid oxidation, and ultimately reducing lipid accumulation, which inhibits energy-intensive processes (Zeqiraj et al., 2009; Sharma et al., 2021). Sirt1, a member of the Sirtuins family, is a class III histone deacetylase dependent on nicotinamide adenine dinucleotide (NAD^+^) (Yue et al., 2016). Its activity is regulated by NAD^+^/NADH, mainly distributed in the nucleus and widely expressed in a variety of mammalian tissues, and participates in the regulation of cell survival and material metabolism through deacetylation of substrates (Tian et al., 2019). However, the biosynthesis of NAD^+^ decreases with increasing age, resulting in a decline in Sirt1 activity (Ramsey et al., 2008). Interestingly, Kim et al. (2015) demonstrated that raising AMPK expression could enhance sirt1 expression. Cantó et al. (2009) found that AMPK was located upstream of Sirt1 and could increase Sirt1 activity by increasing the intracellular AMP/ATP ratio, particularly the level of NAD^+^/NADH (Long et al., 2019). Sirt1 promoted the translocation of LKB1 into the cytoplasm by deacetylating the upstream kinase LKB1 of AMPK, and then increased the phosphorylation and activity of AMPK to regulate lipid metabolism (Lan et al., 2008).

Lipid metabolism is co-regulated by the interaction of various factors such as fatty acid synthase FAS, rate-limiting enzyme ACC for *de novo* fatty acid synthesis, CPT1 for fatty acid oxidation, and transcriptional regulator SREBP-1c, etc. The LKB1-AMPK-sirt1 signaling axis can regulate hepatic lipid metabolism and reduce fat accumulation through one or more of the above factors Figure 1. Previous studies have shown that when the intracellular AMP/ATP ratio increased, LKB1 could promote the phosphorylation of AMPK, which in turn activated sirt1, and ultimately led to deacetylation of downstream target genes, such as PGC-1α, to inhibit the activities of lipid synthesis genes ACC and FAS (Chau et al., 2010; Tian et al., 2019). Meanwhile, in order to regulate lipid metabolism and keep the body functioning normally, activated LKB1 could block the expression and transcription of the adipogenesis gene SREBP-1c and stimulate the expression and transcription of the fatty acid-oxidizing gene CPT1 (Gu et al., 2019). As a result, by controlling lipid metabolism, the LKB1-AMPK-Sirt1 signaling axis could prevent FLD. Additionally, AMPK may be developed as a novel therapeutic target, offering a potential FLD treatment option.

### AMP-activated protein kinase-ACC-carnitine acyltransferase1 axis

It is well known that fatty acid oxidation, which includes mitochondrial and peroxisome β-oxidation, and microsomal ω-oxidation, is crucial for energy production and the recycling of the carbon skeleton, with mitochondrial β-oxidation being primarily responsible for the oxidation of short, medium, and long chain fatty acids (Adeva-Andany et al., 2019). Under the catalysis of relevant enzymes, fatty acids are transferred from the cytoplasm to the mitochondrial matrix and decomposed into acetyl-CoA, which is further completely oxidized by a series of biochemical reactions such as tricarboxylic acid cycle. However, since medium and long-chain fatty acids cannot freely transmembrane into the mitochondria, they need to be completed by CPT1 on the mitochondrial membrane, so the activity of CPT1 is directly related to the oxidation efficiency of fatty acids (Ribas and Vargas 2022). In contrast, the rate-limiting enzyme ACC for *de novo* fatty acid synthesis is mainly responsible for catalyzing the formation of malonyl-CoA from acetyl-CoA, and then using malonyl-CoA as a substrate to synthesize fatty acids (Bence and Birnbaum 2021). Malonyl-CoA is both a precursor of fatty acid synthesis and an allosteric inhibitor of CPT1. In liver cells, AMPK activated can inactivate ACC by phosphorylating it, thereby reducing the synthesis of malonyl-CoA, enhancing the expression of CPT1, and finally reducing fatty acid synthesis and increasing fatty acid oxidation (Tian et al., 2019). García-Villafranca et al. (2008) investigated whether AMPK played a role in the development of ethanol-induced fatty liver. Their results found that chronic ethanol exposure reduced the expression and activity of AMPK and CPT1 in hepatocytes, which was effectively reversed by treatment with the AMPK agonist AICAR. Additionally, in NAFLD, Sharma et al., (2021) also found that Berbamine could activate AMPK in rat liver cells, which then phosphorylates ACC to inactivate it and reduce the generation of fat in liver. Therefore, activation of AMPK may inhibit ACC, reduce malonyl-CoA synthesis, increase CPT1 activity, thereby promoting the oxidation of fatty acids. It is also worth noting that acetyl-CoA, formed by the oxidation of mitochondrial fatty acids, also plays an important role in the production of ketones, which the brain uses primarily as an energy substrate when glucose is scarce (Ribas and Vargas 2022).

### AMP-activated protein kinase-SREBP-1c-lipin-1 axis

The Sterol regulatory element binding protein family (SREBPs), which includes SREBP-1a, SREBP-1c, and SREBP-2, was originally discovered as a transcription factor that transcribed the expression of genes related to fatty acid, TG, and cholesterol metabolism, and is now recognized as the primary regulatory mechanism of intracellular lipid content change (Brown and Goldstein 1997; Bertolio et al., 2019). SREBP-1a and SREBP-1c are two similar proteins that are transcribed by different promoters and encoded by a single gene, with difference only in the N-terminal region (Xu et al., 2013). SREBP-2 is a metabolic gene that activates cholesterol and a member of the liver cholesterol metabolism transcription factor, whereas SREBP-1c is largely expressed in liver tissue and is in charge of controlling the expression of genes associated to fatty acid production (Hampton 2002). SREBP-1c is synthesized on the endoplasmic reticulum and induced, and then transferred to the Golgi apparatus for processing, forming an active mature form. Subsequently, SREBP-1c enters the nucleus and activates adipogenesis genes, participating in the regulation of downstream fatty acid synthesis rate-binding enzyme ACC and fatty acid oxidation rate-binding enzyme CPT1, resulting in liver lipid metabolism disorder (Osna et al., 2017; Ge et al., 2020). One of the three subtypes of the lipin family (lipin-1/2/3), lipidin-1 is a bifunctional protein with both enzymatic and transcriptional regulatory capabilities that regulates intracellular lipid metabolism (Fan et al., 2018). As a Mg^2+^-dependent phosphatidic acid phosphohydrolase (PAP) in the cytoplasm, Lipin-1 mainly catalyzes the conversion of phosphatidic acid to diglycerides on the endoplasmic reticulum membrane, followed by the synthesis of triglycerides and phospholipids (Carman 2019; Reue and Wang 2019). When lipin-1 is transferred to the nucleus, lipin-1 acts as a transcription coactivator affecting the activity of metabolic transcription factors such as Peroxisome proliferator-activated receptor *α* (PPARα) and peroxisome proliferator-activated receptor coactivator 1-alpha (PGC-1α) (Finck et al., 2006; Wang et al., 2021). Their interaction promotes that oxidation of fatty acids and inhibit the activity of SREBP-1 to reduce the synthesis of fatty acids. Notably, when lipin-1 acts as a transcription coactivator, its activity does not appear to require PAP function, but it does require a hydrophobic moxa (LXXIL) downstream of the active site of PAP to mediate the interaction between Lipin-1 and the transcription factor (Finck et al., 2006). Lipin-1 is further divided into two protein subtypes in liver tissue, lipin-1α and lipin-1β. Lipin-1α is primarily distributed in the nucleus of hepatocytes and promotes the expression of fatty acid oxidation genes, whereas lipin-1β is primarily distributed in the cytoplasm and promotes the expression of fatty acid synthesis genes (Hu et al., 2013; Chen et al., 2019b). Chen et al. investigated whether dihydroartemisinin alleviated AFLD by regulating lipin-1 signaling pathway (Chen et al., 2019b). They discovered that long-term ethanol exposure significantly increased the proportion of Lipin1β/α in mouse livers and increased fatty acid synthesis. However, after treatment with dihydroartemisinin and rapamycin, the ratio of Lipin1β/α was significantly reduced. It can be seen that the Lipin1β/α ratio in liver tissue has a direct effect on the stability of liver lipid metabolism.

The AMPK-SREBP-1c axis has been implicated in the regulation of ethanol-induced lipid metabolism disorders, according to research. When AMPK is activated, it can prevent SREBP-1c protein expression from increasing in alcohol-induced acute and chronic hepatic steatosis, restore ACC phosphorylation, and reduce the activity and expression of adipogenesis gene (Li et al., 2018). In addition, AMPK-SREBP-1c axis has been confirmed to play an important role in the upstream of lipin-1 protein and regulate the expression of lipin-1 gene in AFLD. Hu et al. (2012) overexpressed AMPK protein in ethanol-treated AML-12 cells with AMPK agonist (AICAR). They found that the activity of the lipin-1 promoter and its increase in mRNA levels were significantly suppressed. However, when SREBP-1c was overexpressed in the nucleus, the effect was largely opposite. In conclusion, excessive drinking can inhibit AMPK activity and increasing SREBP-1c expression, which promotes the expression of the downstream target protein lipin-1 of AMPK-SREBP-1c, and increases fatty acid synthesis, ultimately aggravating alcoholic liver injury. As a result, AMPK may be a promising target for regulating lipid homeostasis in the body.

### AMP-activated protein kinase-mammalian target of rapamycin axis

Mammalian target of rapamycin (mTOR) is a highly conserved atypical serine/threonine protein kinase in eukaryotic cells (Fekete et al., 2020). Some research showed that mTOR is a member of the phosphoinositide 3-kinase (PI3K) related kinase family, which interacts with multiple proteins to form two complexes with distinct functional and biochemical composition, namely, mTOR complexes 1 (mTORC1) and 2 (mTORC2), which are crucial for controlling cell growth, metabolism, and autophagy (Laplante and Sabatini 2012; Chen et al., 2021). Complexes containing mTOR have different sensitivities to rapamycin, as well as different upstream inputs and downstream outputs. Five parts make up the mammalian mTORC1 complex: mTOR, Raptor, PRAS40 (also known as AKT1s1), DEPTOR, and MLST8 (also known as G-L) (Rabanal-Ruiz et al., 2017). Among them, Raptor is an mTOR regulation-related protein that mainly acts as a scaffold to recruit downstream substrates such as 4E-BP1 and ribosomal S6 kinase (p70S6K1) into mTORC1 complex to be phosphorylated by mTORC1; PRAS40 is an inhibitory subunit of 40 kDa substrate containing proline-rich Akt; MLST8 is a mammalian lethal Sec13 protein eight related to a stable kinase activation ring and a catalytic domain; DEPTOR is an mTOR interaction protein containing a DEP domain (Rabanal-Ruiz et al., 2017; Holczer et al., 2019; Fekete et al., 2020). That mTORC1 is directly regulated by cellular energy and nutritional status, plays an important role in translation and autophagy regulation, and is able to sense the fluctuation of growth factor signals, cellular energy (*via* AMPK), and oxygen levels (Szwed et al., 2021). It is highly sensitive to the inhibition of rapamycin, but not mTORC2, which consists of mTOR, Rictor, mSin1 (MAPKAP1), Protor (PRR5), and mLST8, is primarily responsible for regulating cell survival and cytoskeleton (Holczer et al., 2019; Szwed et al., 2021). A large number of studies have shown that AMPK and mTORC1 are two key factors that regulate autophagy and can promote and inhibit autophagy, respectively. At the same time, AMPK can negatively regulate mTORC1 activity by directly phosphorylating multiple components in the mTORC1 pathway, and then promote cellular Autophagy. For example, 1) AMPK phosphorylates the negative regulatory factor TSC2 upstream of mTORC1 and activates TSC, thereby attenuating the TORC1 pathway, which has tumor suppressive effects (Inoki et al., 2003; Holczer et al., 2019) (Figure 1). 2) Cells negatively regulate mTORC1 activity through AMPK-induced phosphorylation at the Ser722 and Ser792 on Raptor (Gwinn et al., 2008). Notably, research has revealed that AICAR was unable to inhibit the activity of mTORC1 in fibroblasts with TSC deficiency even if Raptor was completely phosphorylated by AMPK, but it inhibited the activity of mTORC1 in TSC-deficient hepatocytes by increasing Raptor phosphorylation (Wolff et al., 2011). It indicated that the cell type and tissue studied were closely related to the involvement of TSC in AMPK-induced regulation of mTORC1. 3) In glucose deficiency, after treatment with AICAR and 2-deoxyglucose (2DG), the p38β-PRAK pathway was activated independently of AMPK, and the activated PRAK directly phosphorylated the Ser130 site on Rheb to down-regulate the expression level of Rheb, thereby inhibiting the activity of mTORC1 (Zheng et al., 2011). It should be noted that in cells treated with AICAR (Inoki et al., 2012): 1) P38β-PRAK-dependent regulation belongs to the persistent inhibition of mTORC1, because the regulation mode of p38β-PRAK-dependent Rheb phosphorylation only occurs after the initial inhibition of mTORC1 activity. 2) TSC2-or Raptor-mediated inhibition of mTOR RC1 is attributed to acute inhibition of mTOR RC1. It has been confirmed that when mTORC1 is inhibited, it will induce autophagy by activating the downstream ULK1 complex, and AMPK can directly phosphorylate multiple sites of ULK1, thus promoting the function of ULK1 expression in autophagy (Kim et al., 2011; He et al., 2020; Licheva et al., 2022).

Autophagy is an intracellular lysosomal degradation system. In mammals, it mainly includes three mechanisms: partner-mediated autophagy, micro-autophagy and macrophage autophagy (Wirawan et al., 2012; Zhao et al., 2022). The autophagy process is mainly divided into the following three steps (Lamb et al., 2013; Zhao et al., 2022): 1) The damaged or redundant cellular components such as proteins or organelles are wrapped to form autophagosomes with a double-layer membrane structure; 2) Autophagosomes fuse with intracellular lysosomes to form autophagosomes; 3) The inclusion is degraded into basic biomolecules by the acidic hydrolase in the lysosome and recycled back to the cytoplasm to realize the reuse of cell components and energy supply. Moreover, as a basic homeostasis mechanism, autophagy plays an important role in the process of various diseases in the human body. It can remove excessive intracellular fat, which is essential to maintain the normal function of cells and regulate lipid balance. On the contrary, if autophagy in adipocytes is impaired, it can lead to cellular lipid accumulation, causing obesity, dyslipidemia, fatty liver and other diseases (Singh et al., 2009; Zechner et al., 2017; Saito et al., 2019). AMPK-mTOR axis plays a very important role in the regulation of lipid metabolism. AMPK may be a potential target for the treatment of FLD. To date, many predecessors have confirmed this view in the scientific research field and clinical practice. For example, caffeine, the main ingredient extracted from coffee, can activate autophagy and accelerate fatty acid oxidation by blocking the mTOR pathway (Sinha et al., 2014). Some scholars have found in the mouse model of NAFLD that inhibiting autophagy can lead to significant lipid accumulation, while promoting autophagy can regulate the use of liver lipids and maintain the body energy metabolism (Tanaka et al., 2016; Sun et al., 2018). The above research showed that the excessive accumulation of lipid droplets in hepatocytes could be degraded by regulating the autophagy process, and its toxic effect on hepatocytes could be reduced, thus inhibiting the further development of NAFLD. In addition, natural products resveratrol and acetylshikonin can improve autophagy function by acting on AMPK-mTOR pathway, and to a certain extent reduce non-alcoholic liver lipid accumulation, hepatic steatosis (Milton-Laskibar et al., 2018; Zeng et al., 2018). Similarly, Guo et al. (2016) found that corosolic acid extracted from leaves of Lagerstroemia speciosa could inhibit mTORC1 by activating AMPK to a certain extent in a rat model of alcoholic liver injury, so that the autophagy activity inhibited by alcohol is restored, and thus the liver function is protected from alcoholic toxicity. Therefore, AMPK-mTOR axis-mediated autophagy plays an important role in improving FLD.

## Fatty liver disease

### Alcoholic liver disease

**FIGURE 2:**
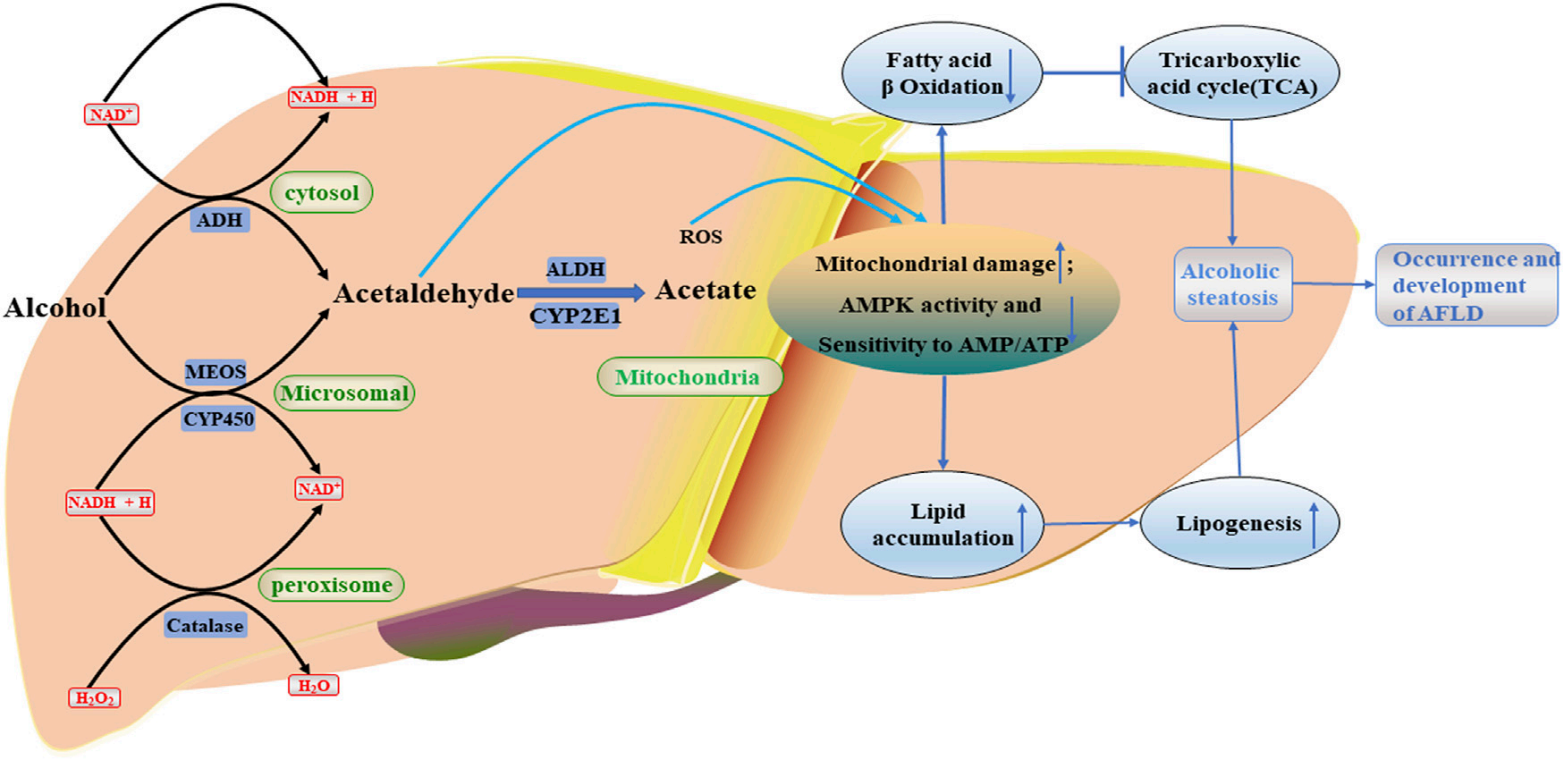
Occurrence and development of AFLD. Alcohol metabolites, including acetaldehyde, will further damage mitochondria, reduce AMPK activity and sensitivity to AMP/ATP, and cause serious lipid accumulation. ROS, reactive oxygen species; CYP2E1, the cytochrome P4502E1; ADH, alcohol dehydrogenase; ALDH, aldehyde dehydrogenase; NADPH, reduced nicotinamide adenine dinucleotide phosphate; NAD^+^, oxidized nicotinamide adenine dinucleotide.

Chronic and excessive drinking can cause severe liver diseases such as AFLD (hepatic steatosis), alcoholism (hepatitis, fibrosis, cirrhosis) and even hepatocellular carcinoma (HCC) (Nagy et al., 2016; Zhou et al., 2022). AFLD, also known as hepatic steatosis, is the initial reaction of chronic and heavy drinking, a stage of alcoholic liver disease that is considered a vicious circle due to abnormal lipogenesis and lipid *β* oxidation, characterized by excessive deposition of fat in hepatocytes (Hsu et al., 2018). Long-term heavy drinking may accelerate the transformation from AFLD to alcoholic hepatitis, it is a more severe inflammatory liver injury characterized by steatosis, hepatocyte inflation, and neutrophil infiltration with or without fibrosis, and may also lead to the development of fibrosis, which is characterized by excessive deposition of extracellular matrix proteins (Altamirano and Bataller 2011; Hyun et al., 2021). The primary biochemical explanation for AFLD is largely based on ethanol metabolism’s capacity to prevent fatty acid oxidation and change the liver’s redox state (Namachivayam and Gopalakrishnan 2021). And studies have shown that ethanol has been found to inhibit fatty acid oxidation and promote lipogenesis by activating SREBP-1c to induce a series of lipase (Bai et al., 2016). The main metabolic mechanisms of alcohol metabolism in the body and its effects are as follows Figure 2:1) Ethanol dehydrogenase and CYP2E1 oxidize ethanol to acetaldehyde, which is further converted to acetic acid by acetaldehyde dehydrogenase, and finally enters the tricarboxylic acid cycle to generate acetyl-CoA (Marmier et al., 2015). 2) A large amount of reactive oxygen species (ROS) is produced during ethanol metabolism, which can reduce antioxidant enzyme activity or promote lipid peroxidation and adduct formation, thereby inhibiting the antioxidant capacity of hepatocytes (Cederbaum 2012). 3) The oxidation of acetaldehyde occurs in the mitochondria of hepatocytes, and the generated acetic acid will induce the generation of a large number of ROS to damage mitochondrial DNA, which will hinder electron transfer in the oxidative respiratory chain, reduce the activity of respiratory chain complex, thereby inhibiting β- Oxidation of fatty acids, increasing the fat content in hepatocytes, accelerating the occurrence and development of FLD (Lieber 2000; Seitz et al., 2018). Additionally, since CYP2E1 has high NADPH oxidase activity, it can stimulate NADPH transport to the mitochondria, increase the generation of ROS, aggravate the damage of mitochondrial DNA, and further inhibit β-oxidation of fatty acids (Cederbaum 2001). Lu et al. (2010) performed evaluation on alcohol-induced chronic liver injury, steatosis and oxidative stress in mice, and they discovered that alcohol caused fatty liver and oxidative stress in wild-type mice, which were somewhat alleviated in CYP2E1 knockout mice, but recovered in humanized CYP2E1 knockout mice. These results indicated that CYP2E1 played an extremely important role in alcohol-induced fatty liver disease and oxidative stress. In both *in vivo* and *in vitro* experiments with alcohol exposure, AMPK activity was downregulated as an adaptive response to alcohol-mediated fatty liver disease and liver sensitivity to changes in AMP/ATP ratio was reduced (García-Villafranca et al., 2008). Therefore, AMPK may be a potential therapeutic target for AFLD. Hsu et al. (2018) found that aqueous extract of Pepino leaf (AEPL) could down-regulate the expression of AMPK downstream adipogenesis gene (SREBPs, ACC, and FAS) by up-regulating AMPK activity, to reduce lipid accumulation and inhibit further ethanol-induced proliferation and fibrosis of C57BL/6J mice hepatocytes. Moreover, Wang et al. (2022b) took SD rats as the research object to explore the protective effect of selenium tea extract on fatty liver induced by high-fat diet/alcohol. They found that after treatment with selenium tea extract, the activities of SREBP1 and FAS proteins in rats with AFLD were inhibited, and the expression of p-AMPK, PPAR-α and CPT1 proteins was significantly increased. At the same time, serum and liver TC and TG levels and serum LDL-C level of AFLD rats were decreased, and serum HDL-C level was increased. The above results indicated that selenium tea extract might reduce lipid deposition and alleviate the metabolic imbalance of AFLD by stimulating AMPK/SREBP1-c/FAS and PPAR-α/CPT1 signaling pathways. In addition, studies have shown the activation of AMPK in AFLD may regulate autophagy of cells through a dual “failure-safe” mechanism. Normally, acute alcohol exposure induces autophagy to alleviate liver injury, whereas chronic alcohol exposure inhibits autophagy and triggers lipid accumulation in the liver (Thomes et al., 2015; Chao et al., 2018). This may be a protective mechanism of rapid response by cells to reduce the damage to themselves caused through adverse external stimulation during acute ethanol exposure. However, when exposed to chronic ethanol, the self-protective autophagic flow and related membrane structures were damaged by long-term ethanol stimulation, which resulted in the inhibition of autophagy. Thus, AMPK may be a potential therapeutic target.

### Non-alcoholic fatty liver disease

NAFLD, as currently the most common liver disease in the world, is a lipotoxic disease characterized by liver steatosis and oxidative stress. The signature feature of NAFLD is the accumulation of intrahepatic triglycerides (TG), and in some patients may develop more serious non-alcoholic steatohepatitis (NASH), cirrhosis or even HCC(Rinella 2015; Schwabe et al., 2020; Gosis et al., 2022). The pathogenesis of NAFLD is very complex. In the pathological condition, it is mainly caused by the increased uptake of fatty acids and *de novo* synthesis of fatty acids by the liver, the imbalance of fatty acid oxidative decomposition, and abnormal lipid output (Ipsen et al., 2018; Rui and Lin 2022). In patients with non-alcoholic hepatitis and animal models, Sunny et al. (2017) discovered that mitochondria had a wide range of structural and functional abnormalities. In other words, the function of mitochondrial respiratory chain is damaged, which changes the balance between anti-oxidation and pro-oxidation mechanisms, and prevents the *β* oxidation of fatty acids, resulting in the increase of non-metabolized fatty acids in cytosol, inducing the production of excessive cytokines and ROS, and thus leading to lipid peroxidation and lipid accumulation (Zhou et al., 2022) Figure 3. Moreover, the ROS and lipid peroxidation products can further disrupt the function of the respiratory chain and form a vicious cycle (Masarone et al., 2018). According to previous studies, it is generally believed that the pathogenesis of NAFLD involves two steps, that is, the so-called “two-hit” model (Chen et al., 2018). Among them, the “first hit” mainly refers to the increase in insulin resistance (IR) due to the intake of high-fat and high-sugar diet or the increase of normal genetic lesions, which in turn causes liver adipocyte infiltration and lipid accumulation (Lim et al., 2010; Chen et al., 2018). The “second hit” means that, based on the first hit, damaged liver cells can cause an increase in inflammatory factors in the body, an imbalance in antioxidant capacity, etc., thereby inducing the combined effects of oxidative stress, endoplasmic reticulum stress and inflammatory cytokines, and finally reversely aggravating the progression of fatty liver, leading to inflammation, necrosis or fibrosis of hepatocytes (Lim et al., 2010; Luo et al., 2022). However, it is worth noting that the application of the “two-hit” theory cannot accurately explain some metabolic disorders and molecular mechanisms in the development of NAFLD. With the deepening of the study, researchers have found that there were many pathogenic factors related to the occurrence and development of NAFLD. These pathogenic factors could interact with each other, or inhibit or promote, to cause repeated blows to the liver. Therefore, the theory of “multiple blows” has gradually been recognized (Buzzetti et al., 2016). Previous studies have found that in the process of the second strike, the AMPK signaling pathway, as an energy-regulating metabolic enzyme, is almost involved in the entire process of the occurrence and development of NAFLD (Dahlhoff et al., 2014) Figure 3. AMPK activation can reduce NAFLD mainly through three ways: inhibiting fat production in liver, increasing fatty acid oxidation in liver and promoting mitochondrial functional integrity in adipose tissue (Smith et al., 2016). The AMPK signaling pathway-related proteins not only effectively balance food and energy consumption, but also promote mitochondrial generation, further fatty acid and glucose metabolism, and effectively reduce the production of ROS and pro-inflammatory cytokines, ultimately inhibiting the development of fatty liver. (Dahlhoff et al., 2014). In recent years, some scholars have established a liver-specific AMPKα1/α2 double-knockout and diet-induced obesity and NAFLD mouse model through genetic engineering technology (Garcia et al., 2019; Zhao et al., 2020). The study found that liver-specific AMPK knockout could aggravate liver lipid accumulation, steatosis, fibrosis and inflammation, and TUNEL staining showed that the number of apoptotic hepatocytes in AMPK knockout mice was significantly increased. The above-mentioned findings provide further support for the role of AMPK as a potential prophylactic and therapeutic target for NAFLD. Lee et al. (2020) found that AMPK-ULK1 (UNC-51-like autophagy-activated kinase 1) axis played a very important role in protecting the lipotoxicity by activating an atypical KEAP1-NFe2L2 pathway dependent on SQSTM1 through their study of mice with SQSMT1 gene knockout. By activating SESN2-mediated AMPK phosphorylation, SQSTM1 can enhance the interaction between AMPK and ULK1, further mediate ULK1 phosphorylation, to induce autophagy in response to fatty liver toxicity, promote the formation of AMPK-ULK1-SQSTM1 complex, cause autophagic degradation of Keap1, and activate the non-normalized Keap1-Nrf2 signaling pathway, thereby protecting the mouse liver from lipotoxicity (Lee et al., 2020). The important clinical significance of this pathway has also been demonstrated in liver samples from human NAFLD patients. Although the pathophysiology of NAFLD is complicated, IR, which virtually involves the entire process, is the most important factor. Therefore, reversing IR is the key for treating NAFLD. AMPK agonists can improve IR by increasing liver lipid synthesis, fatty acid oxidation, and mitochondrial function repair, as previously mentioned. At the same time, a large number of studies have also shown that regulating the production of adipocytokines or the expression of adipocyte-specific genes was one of the most effective approaches to improve IR. For example, adiponectin, as a cytokine secreted by adipocytes, was a specific cytokine of adipocytokines that is most closely related to IR and has the effect of regulating lipid metabolism in the liver (Caselli 2014; Wang et al., 2019). Adiponectin could improve insulin sensitivity when it binds to the adiponectin receptor (AdipoR1/2). Furthermore, adiponectin could activate AMPK activity, which in turn enhances insulin signal transduction of adipose cells, reverses IR, and inhibits the occurrence and development of NAFLD (Wang et al., 2019; Yang et al., 2022). Therefore, AMPK may be a potential new target for the prevention and treatment of NAFLD.

**FIGURE 3:**
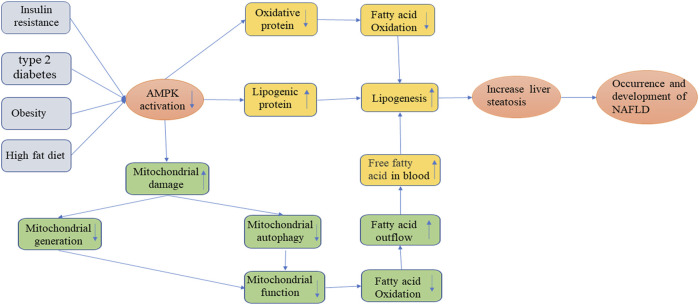
Occurrence and development of NAFLD.

## Research status of fatty liver disease treatment drugs

FLD is a kind of metabolic stress liver injury disease closely related to IR, alcohol, type 2 diabetes, obesity and other diseases. The pathogenesis of FLD is complex, and various factors can act independently or mutually, simultaneously or sequentially, leading to the occurrence and development of AFLD or NAFLD. Even though we have a certain understanding of the occurrence and development of FLD, clinically there is still a lack of the most ideal drug for the treatment of FLD. As AMPK signaling pathway is a key pathway for organisms to resist lipid metabolism disorders. Activation of the AMPK pathway can effectively reduce the accumulation of TG, fatty acids and cholesterol in hepatocytes, slow down the development of fatty liver, and prevent the occurrence and development of liver fibrosis by inducing the normal expression of adipogenesis genes and fatty acid oxidation genes. However, although the current research on FLD has made great progress, due to the multiple intersections of signaling pathways, it has brought great difficulties to further in-depth research on FLD. For example, the latest studies from cultured hepatocytes and animal liver have shown that, on the one hand, Sirt1 could regulate AMPK upstream kinases to activate AMPK; On the other hand, activated AMPK could increase intracellular NAD^+^ level *via* LKB1, and then activate the Sirt1 signaling pathway (Cantó et al., 2009; Bai et al., 2016; Sharma et al., 2021); sirt1 and lipin-1 not only involved in the regulation of inflammatory response, but also participated in the lipid metabolism pathway mediating FLD (You et al., 2015; You et al., 2017); SREBP-1c could directly or indirectly regulate the oxidation of fatty acids, and could also promote the synthesis of fatty acids in cells. Meanwhile, Shen et al. took C57BL/6 mice as the research object to explore whether aucubin could inhibit lipid accumulation and oxidative stress through Nrf2/HO-1 and AMPK signaling pathways (Shen et al., 2019) Figure 1. The results showed that AMPK could alleviate the lipid accumulation and oxidative stress in NAFLD by activating the Nrf2/HO-1 signal axis, thereby preventing the occurrence and development of NAFLD. However, there is no more precise study which has confirmed how the AMPK and Nrf2/HO-1 signaling pathways communicate in FLD, it requires more detailed mechanistic exploration to elucidate.

However, it is noteworthy that in different FLD models, a large number of drugs have been found to exert certain efficacy through the targeted AMPK pathway. For example, endogenous synthetic antioxidant coenzyme Q10 (CoQ10) can be used as AMPK agonist which can inhibit lipogenesis and activate fatty acid oxidation, thereby inhibiting abnormal accumulation of liver lipids and preventing the occurrence and development of NAFLD (Chen et al., 2019a; Gutierrez-Mariscal et al., 2020). CoQ10 can also modulate inflammatory responses through NF-κB-dependent gene expression, as the absence of CoQ10 may result in an increase in proinflammatory factors. Metformin, as an agonist of AMPK pathway, can effectively alleviate IR and liver damage. Besides, it can inhibit the secretion of gluconeogenesis-related enzymes and glucagon-like peptide 1 (GLP-1) as well as glucagon signaling by delaying the absorption of glucose in the gastrointestinal tract (Sunny et al., 2017). In an *in vivo* and *in vitro* experiment, Li et al. (2022) found, by combining computer-aided design analysis, that Atractylenolide III could reduce lipid accumulation by upregulating liver adiponectin receptor 1, activating AMPK pathway, improving the phosphorylation of LKB1 and AMPK, and promoting the expression of fatty acid oxidation proteins CPT1A and PGC1α; At the same time, it could upregulate the activities of antioxidant proteins Sirt3 and Nrf2, inhibit oxidative stress response, and ultimately improve NAFLD (Li et al., 2022). In the *in vitro* experiment of hepatocytes exposed to ethanol, dihydroquercetin was found to significantly increase the activity and phosphorylation of LKB1 and AMPK to inhibit the expression of ACC and SREBP-1c, thereby inhibiting lipid production, promoting fatty acid oxidation and finally reducing fat accumulation (Zhang et al., 2018). In the *in vivo* experiment of mice exposed to ethanol, Kim et al. (2017) found that saponin, an active compound in barley malt extract, could significantly enhance AMPK activity in mice, which in turn regulated target proteins related to lipid metabolism through phosphorylation, and directly inhibit fatty acid synthesis and promote fatty acid oxidation (Kim et al., 2017). It could also degrade the lipid droplets accumulated in hepatocytes by enhancing autophagy, and finally reduce hepatic qualitative change and improve ALFD. Furthermore, many natural AMPK activators and AMPK agonists such as quercetin, triptolide, limonene, and demethyleneberberine can regulate related genes on the AMPK signaling pathway, for example, the expression levels of fatty acid β-oxidation genes (CPT1 and PGC1α) and adipogenesis genes (SREBP, ACC, SCD, and FAS), which regulate lipid metabolism in hepatocytes, inhibit fatty acid production and promote fatty acid β-oxidation to alleviate NAFLD (Qiang et al., 2016; Huang et al., 2021; Wang et al., 2022a; Gnoni et al., 2022). As well as some PPAR agonists currently undergoing II and III clinical trials, such as Elafibrinor and saroglitazar, the former can increase mitochondrial fatty acid oxidation and oxidative phosphorylation, and reduce the activation of Kupffer cells in the liver; the latter can improve the insulin sensitivity of adipose tissues and reduce the flow of fatty acids from adipose tissues to the liver (Konerman et al., 2018). Of course, although the above drugs have a certain effect on the treatment of FLD, they still have defects such as unclear toxic and side effects, optimal dose, unclear mechanism, and lack of a large number of clinical trials. These drugs still need to be further verified by multi-center, large-sample randomized controlled clinical studies before they are officially promoted. In conclusion, improving lipid metabolism, anti-inflammatory and anti-oxidative stress is of great significance for the prevention of FLD, and AMPK may be a potential effective target for the treatment of FLD.

## Outlook

Lipid accumulation is the main manifestation of FLD and plays an important role in the occurrence and development of various liver diseases. AMPK signaling pathway is the key pathway for biological resistance to lipid metabolism disorders. Its mediated expression of adipogenesis gene and fatty acid oxidation gene can effectively maintain the normal lipid metabolism of the body, slow the development of liver diseases, and prevent the occurrence and progression of FLD. Although, up to now, based on a large number of previous studies, we have obtained a certain understanding of the role of related molecules in the AMPK signaling pathway in FLD. Animal models with AMPK gene knockout can be more applied to disease research and help clarify the role of AMPK in the occurrence and development of liver diseases. At the same time, many AMPK agonists, drugs that specifically activate AMPK, and the discovery of small molecule agonists provide tools for further exploration of possible treatments for liver disease. However, most of the studies concerning the treatment of liver diseases with agonists are at the initial stage. Further studies are still needed on the downstream pathways and effector molecules after the activation of AMPK by these agonists, and more high-quality *in vivo* and *in vitro* studies are carried out to clarify the effectiveness of agonists and security. And the effects of AMPK agonists on different liver diseases are reflected more accurately, so as to provide a basis for clinical drug combination in the future. In addition, due to the complex and variable pathogenesis of FLD (AFLD or NAFLD), there is still a lack of recognized targeted drugs for the treatment of the disease in clinic. It is worth noting that recent studies have shown that AMPK activation can promote cholestasis by directly phosphorylating the farnesoid X receptor and inhibiting its activity. For example, the AMPK agonist metformin can induce cholestasis by modulating the farnesoid X receptor (Thomes et al., 2015; Li et al., 2017). Farnesoid X receptor is the earliest bile acid receptor discovered, which can be activated by bile acid, inhibit bile acid synthesis, and promote bile acid transport. Therefore, with the development of molecular biology, in future studies, more use of transgenic technology or specific agonist and inhibitor intervention will help to further reveal the link between the AMPK pathway and the pathogenesis of FLD.
